# Function and Circulation of the Retina and Choroid in Case of Indolent Nonprogressive Multifocal Choroidal Lesions

**DOI:** 10.7759/cureus.61962

**Published:** 2024-06-08

**Authors:** Shinichiro Chujo, Hisashi Matsubara, Hideaki Kawakami, Kumiko Kato, Mineo Kondo

**Affiliations:** 1 Ophthalmology, Mie University Graduate School of Medicine, Tsu, JPN; 2 Ophthalmology, Gifu Municipal Hospital, Gifu, JPN

**Keywords:** choroid, choriocapillaris, retina, multifocal electoretinography, indolent nonprogressive multifocal choroidal lesions

## Abstract

Indolent nonprogressive multifocal choroidal lesions have been reported to be benign choroidal lymphatic lesions that do not affect the visual function. However, as best known, there are no reports on whether these lesions affect the circulation and function of the retina and choroid. We report a case of indolent nonprogressive multifocal choroidal lesions in which retinal images were available to assess the retinal and choroidal circulation and whether it impacted the retinal function. The patient was a 45-year-old man. Swept-source optical coherence tomography (OCT) showed multiple well-defined, uniform, hyporeflective cavernous lesions in the choroidal layer. Then a diagnosis of indolent nonprogressive multifocal choroidal lesions was made based on the similarity of the features with those reported. OCT angiography showed no blood flow signals in the lesions and reduced blood flow signals in the choroid and choriocapillaris directly above the lesions. Fundus autofluorescence showed retinal pigment epithelial damages that were colocalized with the choroidal lesions. We then performed static visual field testing and multifocal electroretinography (mfERG). The static visual field test showed no decrease in sensitivity in the entire visual field, and mfERG showed no decrease in the amplitudes or implicit times indicating normal retinal function. In indolent nonprogressive multifocal choroidal lesions, the photoreceptor function is preserved but a mild retinal pigment epithelium disorder is present. Thus, the follow-up examinations of indolent nonprogressive multifocal choroidal lesions should include retinal function tests.

## Introduction

Indolent nonprogressive multifocal choroidal lesions are benign choroidal lymphatic lesions that were described by Carroll et al. [1] Indolent nonprogressive multifocal choroidal lesions are multifocal. They are flat, uniform, hyporeflective lesions in the choroidal layer. They are clustered in the posterior pole and are most seen in the left eye of middle-aged men. The frequency of occurrence among ethnic groups has not been reported.

Long-term follow-up has shown that indolent nonprogressive multifocal choroidal lesions do not affect the visual function [1,2]. It has also been reported that choroidal lesions may not involve the retina over long periods of observation [3]. However, it is not clear how the indolent nonprogressive multifocal choroidal lesions affect the hemodynamics and function of the choroid and retina. To determine these characteristics, we examined a case of indolent nonprogressive multifocal choroidal lesions in which detailed retinal and choroidal images were available to assess the circulation and function of the retina and choroid.

## Case presentation

The patient was a 45-year-old Japanese man who had no visual symptoms. However, during an annual general checkup, a fundus examination revealed abnormalities in his left eye, and he was referred to an ophthalmologist. There were no reports of any ocular abnormalities in his medical history, family history, or life history. There is no history of systemic disease. His best-corrected visual acuity was 20/20 in both eyes. He has a myopia of about -3.5D. In this case, the axial length was not measured.

The intraocular pressure was 11 and 12 mmHg in the right and left eyes. Slit-lamp examination showed no abnormal findings in the anterior chamber or optic media. 

**Figure 1:**
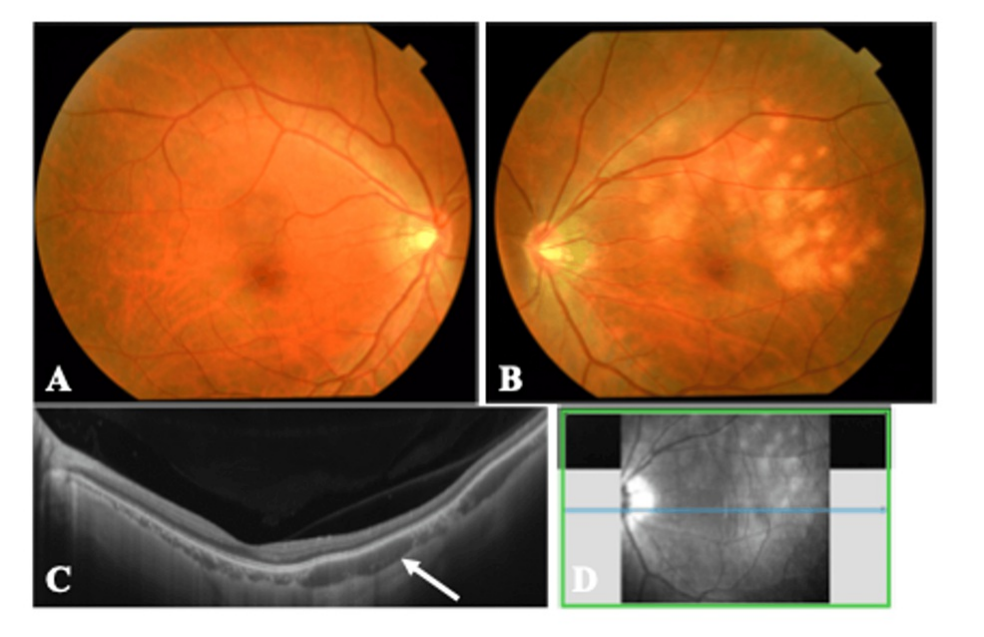
Fundus photographs and optical coherence tomographic image of the patient with indolent nonprogressive multifocal choroidal lesions. A: Fundus photographs showing that the right eye was normal. B: In the left eye, numerous yellowish-white lesions can be seen in the posterior pole and midperiphery. C: Swept-source OCT examination of the lesions revealed many well-defined, uniform, hyporeflective cavity-like lesions in the choroid (white arrow). D: The en face image of swept-source OCT. OCT: Optical coherence tomography

First, a fundus examination was performed to clarify the fundus lesions noted, which revealed a yellowish-white lesion in the choroid (Figures 1A, 1B). To further investigate the lesion more closely, swept source-optical coherence tomography (OCT) was performed, which revealed multiple cavernous choroidal lesions (Figures 1C, 1D). 

**Figure 2:**
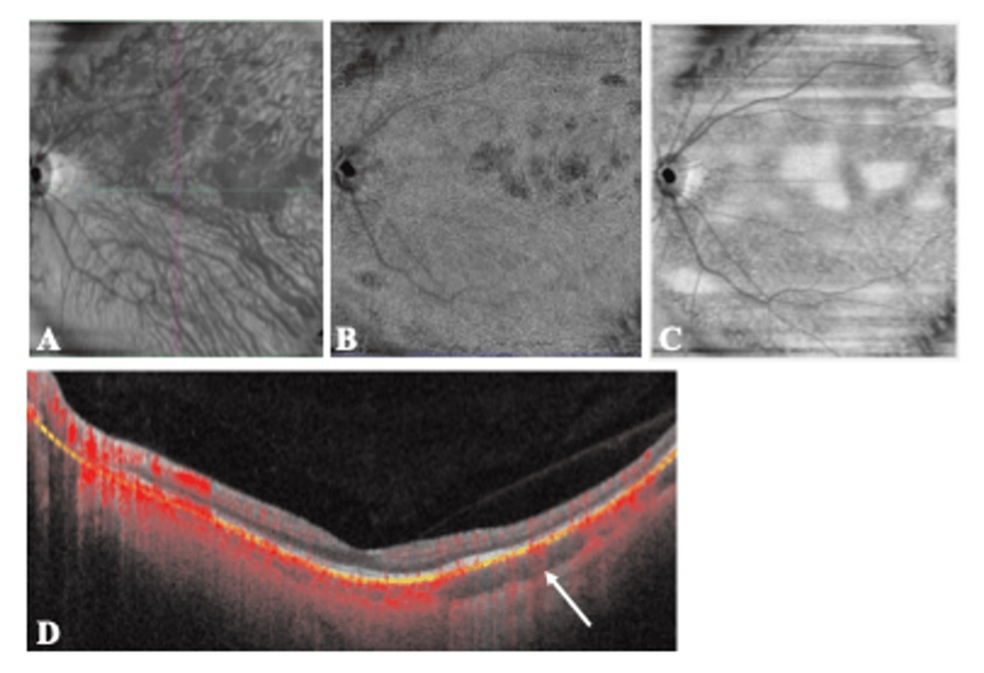
OCTA images (12×12) of the eye of the patient with indolent nonprogressive multifocal choroidal lesions. A: The OCTA image shows reduced blood flow in the choroid. B: Blood flow in the choriocapillaris at the lesion site is lower than in the surrounding area. C: In the structure of choriocapillaris, the lesion is deficient compared to the surrounding structures. D: The image shows that the blood flow signals in the choroidal lesion are reduced. OCTA: Optical coherence tomography

Optical coherence tomography (OCTA) was performed to evaluate the circulatory dynamics near the lesion. OCTA showed reduced blood flow in the choroid and choriocapillaris directly above the lesions (Figures 2A-2C) and no blood flow signals in the lesions (Figure 2D).

**Figure 3:**
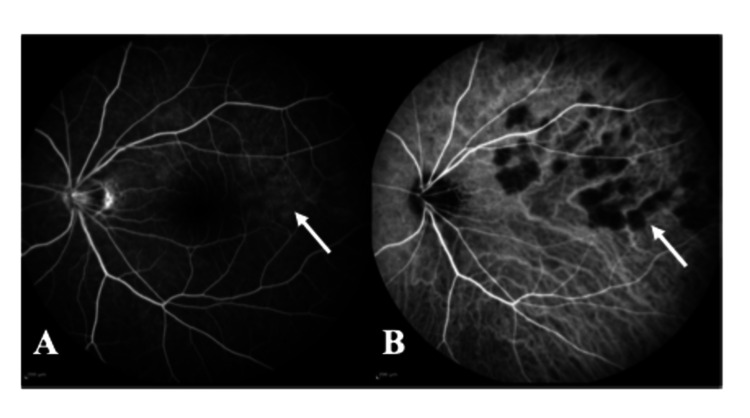
Fluorescein and indocyanine green angiography of the patient with indolent nonprogressive multifocal choroidal lesions. A: Fluorescein angiography reveals hyperfluorescence in an area consistent with choroidal lesions (white arrow). B: Indocyanine green fundus angiography shows hypofluorescent findings consistent with the lesions (white arrow).

Fluorescein angiography and indocyanine green fundus angiography were also performed to examine retinal circulation. Fluorescein angiography showed staining in areas consistent with the choroidal lesions (Figure 3A). Indocyanine green fundus angiography showed hypofluorescence indicated by the white arrow (Figure 3B). In the second course of indocyanine green angiography, those lesions were not stained and not accompanied by choroidal vascular hyperpermeability.

**Figure 4:**
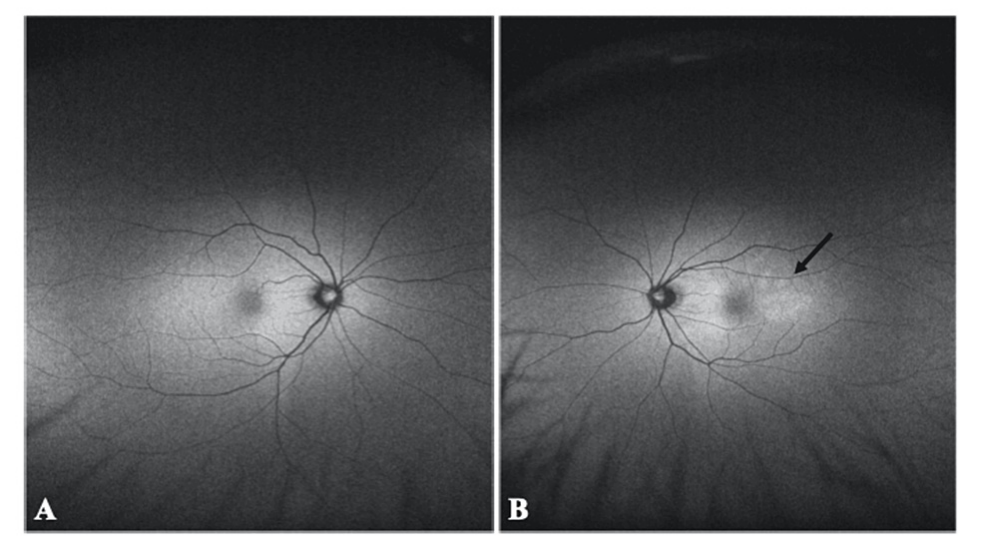
Fundus autofluorescence images of the right and left eyes of the patient with indolent nonprogressive multifocal choroidal lesions. A: Fundus autofluorescence is normal in the right eye. B: Hyperautofluorescence findings in the left eye in areas consistent with the choroidal lesions. (black arrow)

Fundus autofluorescence (FAF) was also performed to investigate retinal dysfunction in the lesion. FAF showed hyperautofluorescence in areas consistent with the choroidal lesions (Figures 4A, 4B). 

Then blood tests were also performed to detect systemic abnormalities. Systemic lymphoma was not suspected. Rapid plasma reagin, angiotensin-converting enzyme, and HLA-A29 were negative.

**Figure 5:**
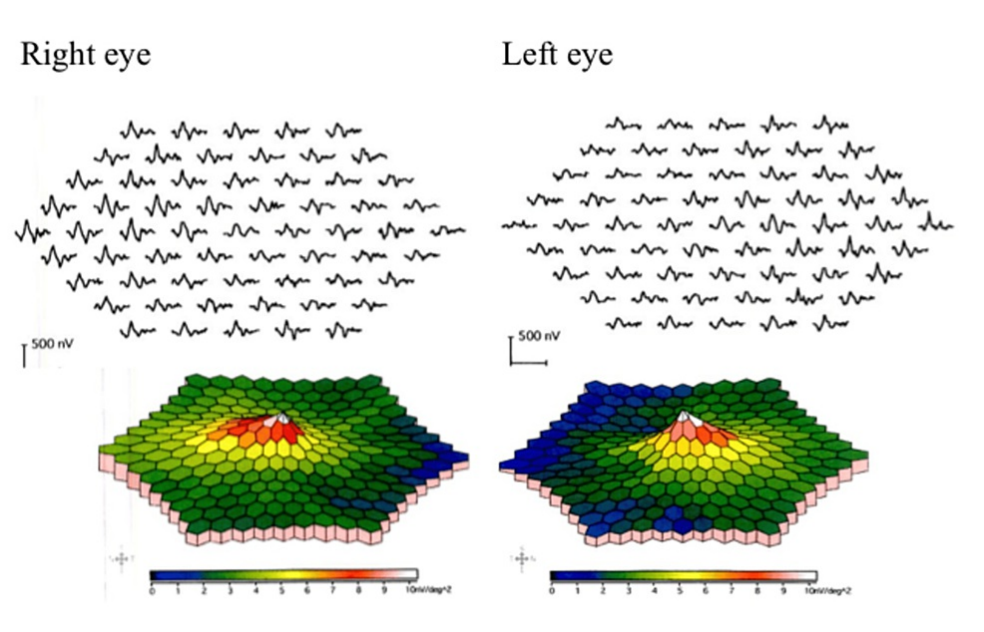
Multifocal electroretinography (mfERG) findings in the patient with indolent nonprogressive multifocal choroidal lesions. mfERG showed no decrease in amplitude or implicit times compared to the same region in the fellow eye.

After confirming the absence of systemic abnormalities, the diagnosis of indolent nonprogressive multifocal choroidal lesions was made based on the similarity of the features with those reported [1,2]. Based on the results of FAF and OCTA, we suspected that this patient may have concurrent retinal pigment epithelium (RPE) dysfunction. We then performed static visual field testing and multifocal electroretinography (mfERG). The static visual field test showed no decrease in sensitivity (Figure 5).

**Figure 6:**
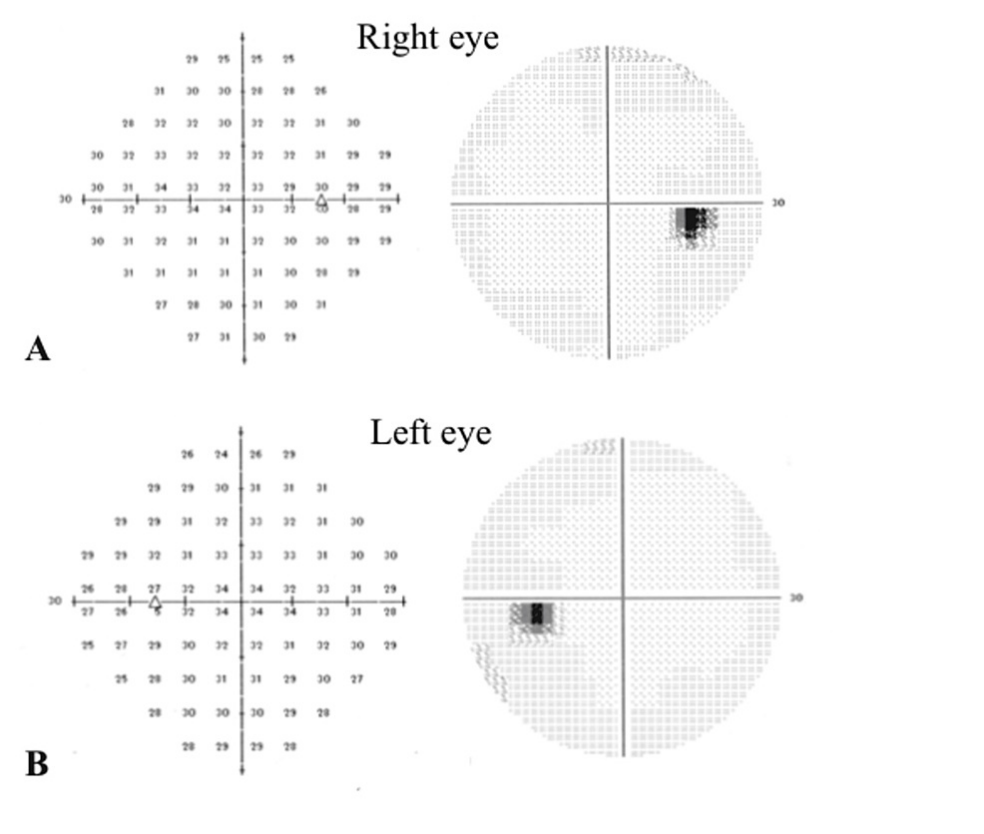
Visual fields of the patient with indolent nonprogressive multifocal choroidal lesions. Static visual fields show no loss of sensitivity in the right and left eyes.

mfERG showed no decrease in amplitude or implicit times compared to the same region in the fellow eye indicating normal retinal function (Figures 6A, 6B).

The patient then requested a referral to a nearby hospital. His vision and visual field remained unchanged and normal for about one year of follow-up.

## Discussion

This report showed a mild dysfunction of the RPE by performing detailed analyses of the images of the retina and choroid in a case of indolent nonprogressive multifocal choroidal lesions.

We hypothesized from the OCTA results that the choroidal lesions would compress the choroid and choriocapillaris resulting in complicated RPE dysfunction. In support of our hypothesis, a similar mechanism of RPE dysfunction was presented by Ali et al. for choroidal nevi using OCTA. Ali et al. reported that a choroidal nevus reduced the blood flow in the choroid and choriocapillaris superior to the nevus causing an associated ischemia of the outer retinal layers [4]. Similar to these mechanisms, it is suggested that RPE dysfunction may occur in indolent nonprogressive multifocal choroidal lesions.

We also hypothesized that there was another photoreceptor dysfunction complicated by the RPE disruption. Therefore, we tested for photoreceptor dysfunction using visual field testing and mfERG to validate our findings. mfERG is able to record many focal electroretinograms of the posterior pole in one stimulation session and in a few minutes based on visual stimuli called multilocal inputs. It can also detect abnormalities in the outer retinal layers [5]. In the diagnosis of acute zonal occult outer retinopathy and occult macular dystrophy, mfERG has been useful in detecting abnormalities in the outer retinal layers [6,7].

We hypothesized that if photoreceptor damage is present in indolent nonprogressive multifocal choroidal lesions, the mfERG would show a decrease in the amplitude consistent with a lesion. However, there is no obvious decrease in amplitude compared to the same region in the fellow eye. There was also no decrease in the threshold in static visual field testing. These results suggested that the photoreceptor function is preserved in indolent nonprogressive multifocal choroidal lesions despite the mild RPE impairment. However, the possibility of photoreceptor damage cannot be ruled out if the duration of the course of the disease or the degree of pressure drainage caused by the lesions is severe. Thus, clinicians must carefully monitor the progress of the disease.

There are several limitations in this report. The first limitation is that we had not performed an electrooculogram (EOG). We should have performed EOG to analyze the RPE function. A second limitation is the sample size. Multiple case studies are needed to evaluate our results.

## Conclusions

We studied one case of indolent nonprogressive multifocal choroidal lesions and analyzed the circulation and function of the choroid and retina. It was found that the photoreceptor function was preserved, but mild RPE impairment was present. Therefore, follow-up of indolent nonprogressive multifocal choroidal lesions should include tests for the integrity of the circulation and function of the choroid and retina. And the clinician should then follow up with consideration of the effect on retinal function.
